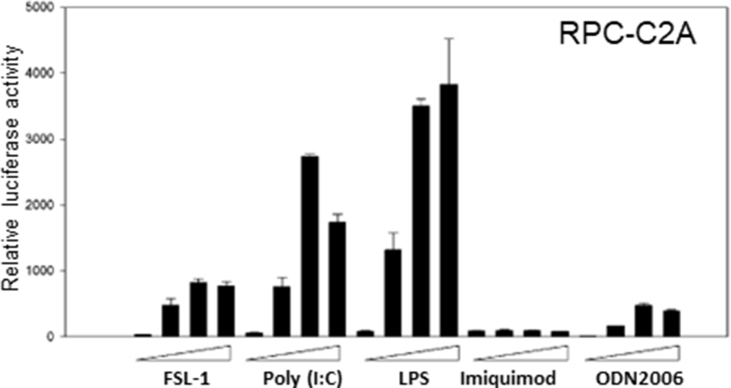# Human adenovirus oncolytic properties and the inhibitory role of E4 orf4 and E4 orf6/7 on endogenously activated NF-κB

**DOI:** 10.1016/j.bbrep.2023.101616

**Published:** 2023-12-22

**Authors:** Anran Wang, Kazuki Uchida, Atsuro Yokoyama, Fumihiro Higashino, Motoaki Yasuda

**Affiliations:** a Department of Oral Functional Prosthodontics, Division of Oral Functional Science, Faculty of Dental Medicine and Graduate School of Dental Medicine, Hokkaido University, Japan; b Department of Oral and Maxillofacial Surgery, Division of Oral Pathobiological Science, Faculty of Dental Medicine and Graduate School of Dental Medicine, Hokkaido University, Japan; c Department of Medical Management and Informatics Medical Management and Informatics Clinical Engineering, Hokkaido Information University, Japan; d Department of Oral Molecular Microbiology, Division of Oral Pathobiological Science, Faculty of Dental Medicine and Graduate School of Dental Medicine, Hokkaido University, Japan

**Keywords:** Adenovirus, E4 orf4, E4 orf6/7, NF-κB, TLR2

## Abstract

Human adenovirus is a promising tool for cancer therapy as an oncolytic virus. To predict which region of the oncolytic adenovirus E4 gene could be deleted, we investigated the relationship between the E4 proteins and NF-κB. Here, we report that TLR2-dependent NF-κB activation in Ad5-infected cells was significantly inhibited 24 h post-infection. Among the six E4 proteins, E4 orf4 and E4 orf6/7 exhibited notable suppressive effects on NF-κB activation. However, only E4 orf4 was co-immunoprecipitated with the RelA protein, also known as p65. It appears likely that E4 orf6/7 represses NF-κB activation via E2F-dependent pathways. Our results suggest that both E4 orf4 and E4 orf6/7 are novel inhibitors of NF-κB activation. The inhibition of endogenous NF-κB activation by E4 proteins during the late phase of infection also appears to elucidate the previously reported suppression of E1A expression in the late phase of infection. These redundant suppressive effects of E4 orf4 and E4 orf6/7 on NF-κB suggest that these proteins may play a major role in the anticancer properties of oncolytic adenovirus.

## Highlights

• Ad-induced TLR2-dependent NF-κB activation is significantly inhibited 24 h post-infection by E4 proteins.
• E4 orf4 and E4 orf6/7 suppress NF-κB activation via independent pathway at the human adenovirus late infection stage.
• E4 orf4 associates with RelA.
• E2F4 associate with RelA and E2F4/RelA complex is sequestered in cytosol.

## 1 Introduction

Human adenovirus type 5 (Ad5) serves as the backbone for oncolytic viruses. The Ad5 genome comprises five early transcription units (E1A, E1B, E2, E3, and E4) [1,2], along with a late transcription unit that encodes five late proteins (L1 through L5) [3]. During the early phase of infection, E1A and E4 proteins become detectable approximately 12 h after infection [4]. While E1A and E1B are extensively studied genes and are essential for Ad5 replication, E4 genes also play a crucial role in replication [5]. There are six detectable E4 proteins: E4 orf1, E4 orf2, E4 orf3, E4 orf4, E4 orf6, and E4 orf6/7 [6]. Ectopic expression of these E4 genes has significant effects on mRNA export [7], cell cycle regulation [8], and apoptosis [9,10], with some contribution to oncogenic transformation [11].

Ad5 infection triggers a robust innate immune response via toll-like receptor (TLR) sensing, including the activation of vascular endothelial cells and platelets, as well as the production of inflammatory cytokines [12]. TLR2 and 9 have been reported to play a role in the responses in Ad-infected mice [13].The canonical activation of NF-κB plays a major role in these responses. There were several conflicting reports of recombinant Ad5 that lack the whole E4 region. Gao et al. reported that both E1 and E4 deletions may have advantages in terms of safety and efficacy for liver-directed gene therapy, which was accompanied by reduced apoptosis, toxicity, and blunted host immune responses [14]. Lusky et al. reported that deletion of the adenovirus E4 genes did not extend the in vivo persistence of the transduced cells and did not reduce the antivirus immune response [15]. The history of creating E4-deleted Ads aimed at inserting larger genes into limited gene space in Ad. In recent years, we have been working on developing tumor-specific oncolytic viruses [[16], [17], [18]]. E4 orf6-modified Ad is a promising candidate for oncolytic virus development. Therefore, it is important to clarify the involvement of other E4 genes in NF-κB activation, which is thought to play a role in cell survival. This will help determine whether deleting additional regions of E4 is more effective in creating more potent oncolytic Ads. In the present study, we demonstrated that Ad E4 orf4 and orf6/7 exhibited redundant suppressive effects on TLR2-mediated NF-κB activation via independent pathways.

## 2 Materials and Methods

### 2.1 Reagents and antibodies

The mycoplasmal lipopeptide FSL-1 was prepared as described previously [19]. The mouse monoclonal anti-hemagglutinin (HA) antibody (6E2) and anti-Myc antibody (9B11) were obtained from Cell Signaling Technology (Danvers, MA 01923, USA). The mouse monoclonal anti-βActin (AC-15) was obtained from Santa Cruz Biotechnology (Santa Cruz, CA, USA).The rabbit polyclonal anti-RelA antibody were purchased from IBL (Nagoya, Japan). RSA3 (anti N-terminal E4 orf6 monoclonal antibody) was generously provided by Prof. Shenk (Princeton University, NJ, USA). The anti-E4 orf4 polyclonal antibody was obtained by immunizing rabbits with the synthetic peptide corresponding to the 103rd to 114th amino acids of E4 orf4.

### 2.2 Cells, plasmids, and viruses

H1299, HEK293, 3Y1, NIH3T3, and RAW264.7 were obtained from Riken Cell Bank (Tsukuba, Japan). The rat fibroblast RPC-C2A was provided by Prof. Kasugai (Tokyo Medical and Dental University, Tokyo, Japan). HEK293/TLR2 is a stable transfectant of HEK293 cells expressing TLR2. All cell lines were maintained in DMEM supplemented with 10 % heat-inactivated FCS (Sigma, St. Louis, MO, USA). pcDNA TLR1, TLR2, and TLR6 have been described previously [20]. TLR3 was generated by PCR from cDNAs derived from THP-1 cells and cloned into a pEF vector (Invitrogen, Carlsbad, CA, USA). Human TLR4 and TLR9 were purchased from InvivoGen (San Diego, CA, USA). Human Ad5 (wt300) was provided by Prof. Shenk (Princeton University, NJ). Ad LacZ was provided by Prof. Yamashita (Sapporo Medical University, Japan). Non-tagged human RelA (derived from H1299) was cloned into pcDNA3 (Invitrogen, Carlsbad, CA, USA). The NF-κB responsive element (derived from human VCAM), E1 promoter, and E2A promoter of human Ad5 were cloned into the luciferase reporter plasmid pGL3 basic or pGL 4.12 (Promega, Madison, WI, USA). mRNA was extracted from each E4 orf in Ad-infected H1299 cells. Subsequently, reverse transcription was performed, and the resulting cDNA was cloned into the pcDNA3.1 vector with a 5’ HA tag (YPYDVPDYA).

The wild-type E4 orf4 sequence was modified using PCR-based mutations. Specifically, the sequence TATTTC at positions 34072–34077 in the adenovirus type 5 (Ad5) genome (reference sequence: AY33993) was replaced with GAATTC, resulting in a mutant known as E4 orf4 88/89. Additionally, an insertion of ATTC was introduced between the 34015th and 34016th nucleotides, creating a mutant designated as E4 orf4CT. The amino acid sequences resulting from these mutations are detailed in Fig. 3A.

Wild type of HA orf 6/7 and C-terminal truncated mutant (delta 47) were described previously [10]. E2F1 and E2F4 were generated by PCR from cDNAs derived from H1299 and cloned into pcDNA3.1(-) myc vector (Invitrogen, Carlsbad, CA)

### 2.3 Luciferase reporter assay

Cells were plated at 1 × 10^5^ cells per well in 24-well plates a day before transfection. The cells were transiently transfected by the Lipofectamine 2000 reagent (Invitrogen, Carlsbad, CA, USA) with 75 ng of Luciferase reporters, 7.5 ng pGL 4.72 (Promega, Madison, WI, USA), and 300 ng of each expression plasmid. At 24 h after transfection, the cells were stimulated with FSL-1 (0.1–10 nM) or infected with adenoviruses (wt300 or Ad LacZ) in the absence of FBS, and luciferase activity was measured in different time seriese using the Dual-Luciferase Reporter Assay System (Promega, Madison, WI, USA) according to the manufacturer's instructions. Each luciferase activity was measured at least three times, and significance was assessed by the Student's *t*-test.

### 2.4 Detection of E4 proteins

HEK293 or RPC C2A cells were plated at 1 × 10^5^ cells per well in 24-well plates a day before infection. The cells were infected with adenoviruses (wt300 or Ad LacZ) at a multiplicity of infection (MOI) of 20 plaque-forming units (pfu) per cell. After 24 h post-infection, the cells were lysed, and total lysates were used for Western blotting. E4 orf4, E4 orf6, and E4 orf6/7 were detected by polyclonal anti-E4 orf4 antibody or RSA3, employing a chemiluminescence detection system from Amersham Pharmacia Biotech (USA).

### 2.5 Immunoprecipitation

One million HEK293 cells were seeded onto 10-cm dishes one day prior to transfection. The cells were transiently transfected with a total of 20 μg of plasmids. After 48 h post-transfection, the cells were lysed using a buffer containing 500 mM NaCl, 50 mM Tris-Cl (pH 8.0), and 0.1 % Nonidet P-40, supplemented with protease inhibitor cocktails from Sigma (St. Louis, MO, USA) at the recommended concentration by the manufacturer. Immunoprecipitation of proteins was carried out using either an anti-Myc or anti-HA antibody, and the resulting samples were subjected to Western blot analysis using a chemiluminescence detection system from Amersham Pharmacia Biotech (USA).

### 2.6 Confocal laser scanning microscopy

HE1299 cells were transiently transfected with E2F4-GFP plasmids and either DsRed-RelA or DsRed-p50 plasmids. After 48 h of incubation, the medium was removed, and the cells were fixed with 4 % paraformaldehyde for 5 min at room temperature. Following fixation, the cells were washed with PBS and mounted using PermaFluor medium (Beckman Coulter, Fullerton, CA). All samples were examined using a confocal laser microscope (LSM510; Carl Zeiss, Tokyo, Japan).

### 2.7 Statistical analysis

Comparisons were made using an unpaired Student's *t*-test. Data in bar graphs are represented as mean ± standard deviation (S.D.), with significance displayed as follows: NS, not significant; *, p < 0.05; * *, p < 0.01. ; ***,p < 0.001.

## 3 Results

### 3.1 Adenovirus infection induces NF-κB activation via TLR2

**Fig. 1:**
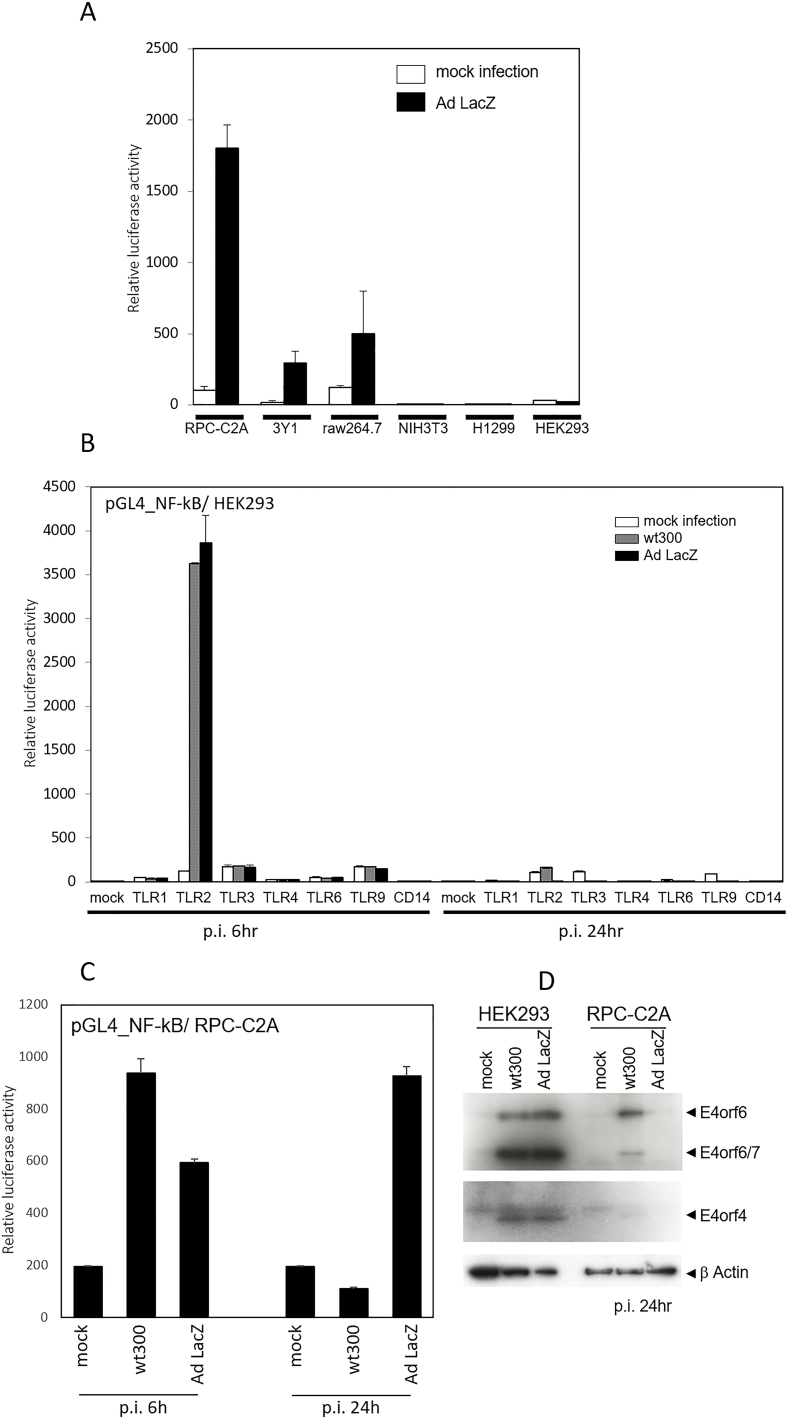
Cell type-specific NF-κB activation upon human adenovirus (Ad) infection. A: The indicated cells were transfected with an NF-κB luciferase reporter plasmid together with a pGL4.72 plasmid, as described in the Materials and Methods section. Transfected cells were infected with Ad wt300 (20 PFU/cell) and incubated for 6 h and lysed, and luciferase activity was measured using the Dual-Luciferase Reporter Assay System. Error bars represent standard deviations for triplicate wells in a single experiment; data are representative of three independent experiments. B: HEK293 cells were transfected with an NF-κB luciferase reporter plasmid together with a pGL4.72 plasmid and the indicated effector plasmids, followed by infection with the indicated Ad (m.o.i = 10 PFU/cell) and incubated for 6 or 24 h. Error bars represent standard deviations for triplicate wells in a single experiment; data are representative of three independent experiments. C: RPC-C2A cells were transfected with an NF-κB luciferase reporter plasmid together with a pGL4.72 plasmid, followed by infection with the indicated Ad (m.o.i = 10 PFU/cell) for the indicated periods. Luciferase assay was performed as described above. Data are representative of three independent experiments. D: HEK293 and RPC C2A cells were infected with indicated adenovirus for 24 h. Subsequently, the cells were lysed, and the corresponding lysates were subjected to Western blotting using RSA3 antibodies (anti-N-terminal region of both E4 orf6 and orf6/7) and anti-E4 orf4 rabbit serum. The lowest row depicts anti-β Actin staining, indicating that equal volumes of cell extracts were analyzed. Endogenous E1A of HEK293 is sufficient for the expression of E4 orf4, 6, and 6/7 with Ad LacZ.

**Fig. S1:**
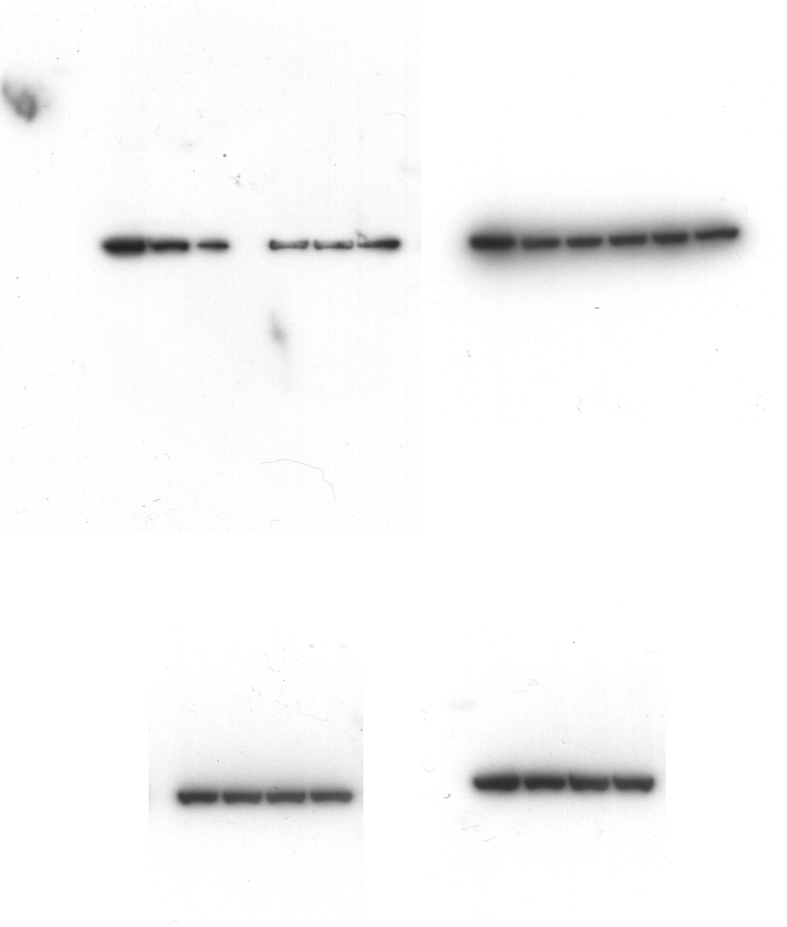


To confirm endogenous NF-κB activation upon Ad infection, several cell lines (rat, RPC-C2A and 3Y1; mouse, NIH3T3 and RAW264.7; and human, H1299 and HEK293) were transfected with the NF-κB luciferase reporter plasmid and examined for NF-κB activation 6 h after infection with wild-type Ad5 (clone wt300) at an m.o.i. of 20 PFU/cell. NF-κB -reporter activation was observed in RPC-C2A, 3Y1, and RAW264.7 cells but not in NIH3T3, HEK293, and H1299 cells (Fig. 1A). These results indicated that the endogenous innate immune system could recognize human Ad particles. Our pilot study demonstrated that RPC-C2A cells expressed several Toll-like receptors (TLRs) and were sufficient to recognize synthetic agonists (supplemental Fig. 1).

To further investigate which TLR(s) are involved in NF-κB activation by Ad5, HEK293 cells were transfected with TLR1, TLR2, TLR3, TLR4, TLR6, TLR9, or CD14 and examined for NF-κB activation 6 h after Ad5 wt300 or Ad LacZ infection. The TLR2 transfectants and, to a lesser extent, the TLR3 and TLR9 transfectants induced NF-κB activation; however, the TLR1, TLR4, TLR6, and CD14 transfectants did not (Fig. 1B). It is likely that TLR2 plays a major role in the recognition of Ad infection in the immediate early phase of infection (within 6 h of infection). Unexpectedly, the TLR2 transfectants did not indicate NF-κB activation 24 h after infection with either wt300 or Ad LacZ. Because HEK293 cells are permissive for Ad multiplication, the amount of virus particles present is much larger at 24 h post infection than the earlier phase of infection (6 h post infection). These observations raise the question of whether these significant differences in NF-κB activation between the short (6 h) and longer (24 h) infection periods are merely a reflection of the pathological condition of Ad-infected HEK293 cells.

To answer this question, the same experiment was performed with the non-permissive cell line RPC-C2A. As shown in Fig. 1C, Ad LacZ showed no suppressive effect at 24 h after the initial infection in RPC-C2A. It is likely that TLR2-dependent NF-κB activation, recognition of Ad particles, continues for at least 24 h and that the activation is suppressed only by wild-type Ad in non-permissive rat cell line, RPC-C2A. Because HEK293 cells express endogenous E1A and E1B, several E4 proteins are expressed at late infection phase (post infection 24 h) even in Ad LacZ-infected HEK 293 cells (Fig. 1D). Therefore, we propose the hypothesis that some E4 oncogene(s) driven by E1 genes are involved in NF-κB suppression.

### 3.2 Association of adenoviral E4 orf4 with RelA

**Fig. 2:**
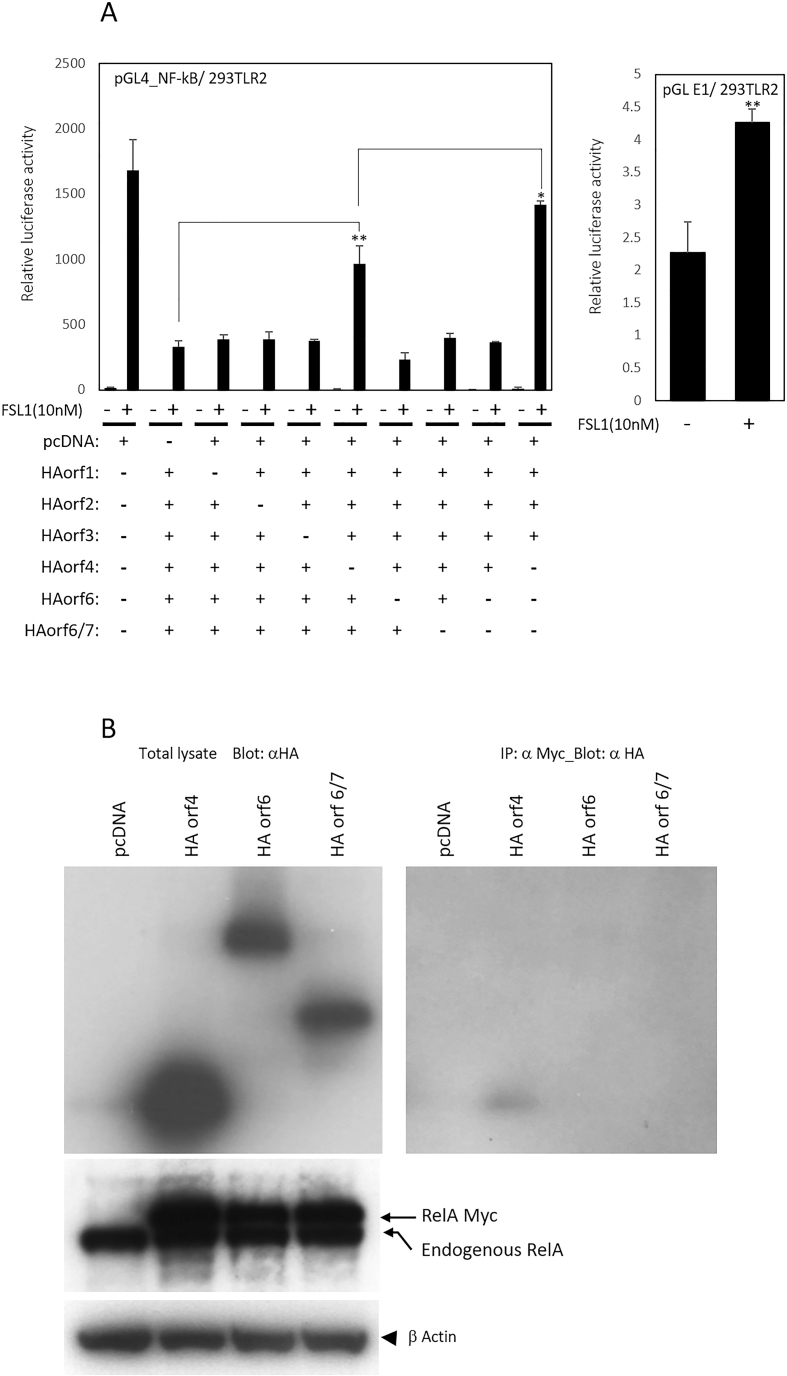
Human adenovirus E4 orfs suppresses the TLR2-dependent NF-κB activation. A: 293TLR2 cells were transfected with an E1 luciferase reporter plasmid or NF-κB luciferase reporter plasmid together with a pGL4.72 plasmid and the indicated effector plasmids, followed by 6 h incubation with FSL-1 (final 10 nM) containing serum free medium. Error bars represent standard deviations for triplicate wells in a single experiment; data are representative of three independent experiments. Comparisons were made using an unpaired Student's *t*-test. Results are represented as mean ± S.D. Statistical significance is displayed as: *, p < 0.05; * *, p < 0.01. B: HEK293 cells were transfected with the combination of Myc tagged hRelA and indicated HA orfs. Cells were incubated for 48 h and lysates were immunoprecipitated with anti-Myc antibody and blotted with anti-HA antibody. Only HA orf4 was co-immunoprecipitated with over expressed myc-tagged RelA protein. Exogenously expressed myc-tagged RelA has a larger molecular weight than endogenous RelA. The lowest row exhibits anti-β Actin staining.

In Ad-infected cells, at least six different E4 proteins (orf1, orf2, orf3, orf4, orf6, and orf6/7) are expressed. To narrow down the list of candidate E4 proteins, each gene was cloned into plasmids containing an HA tag. The HA-tagged E4 orfs and the NF-κB reporter gene were then transfected into 293TLR2 cells (TLR2-expressing HEK293 cells), as indicated by the combinations shown in Fig. 2A, followed by stimulation with a TLR2 agonist. Fig. 2A clearly indicates the involvement of E4 orf4 and, to some extent, E4 orf 6/7.

**Fig. 3:**
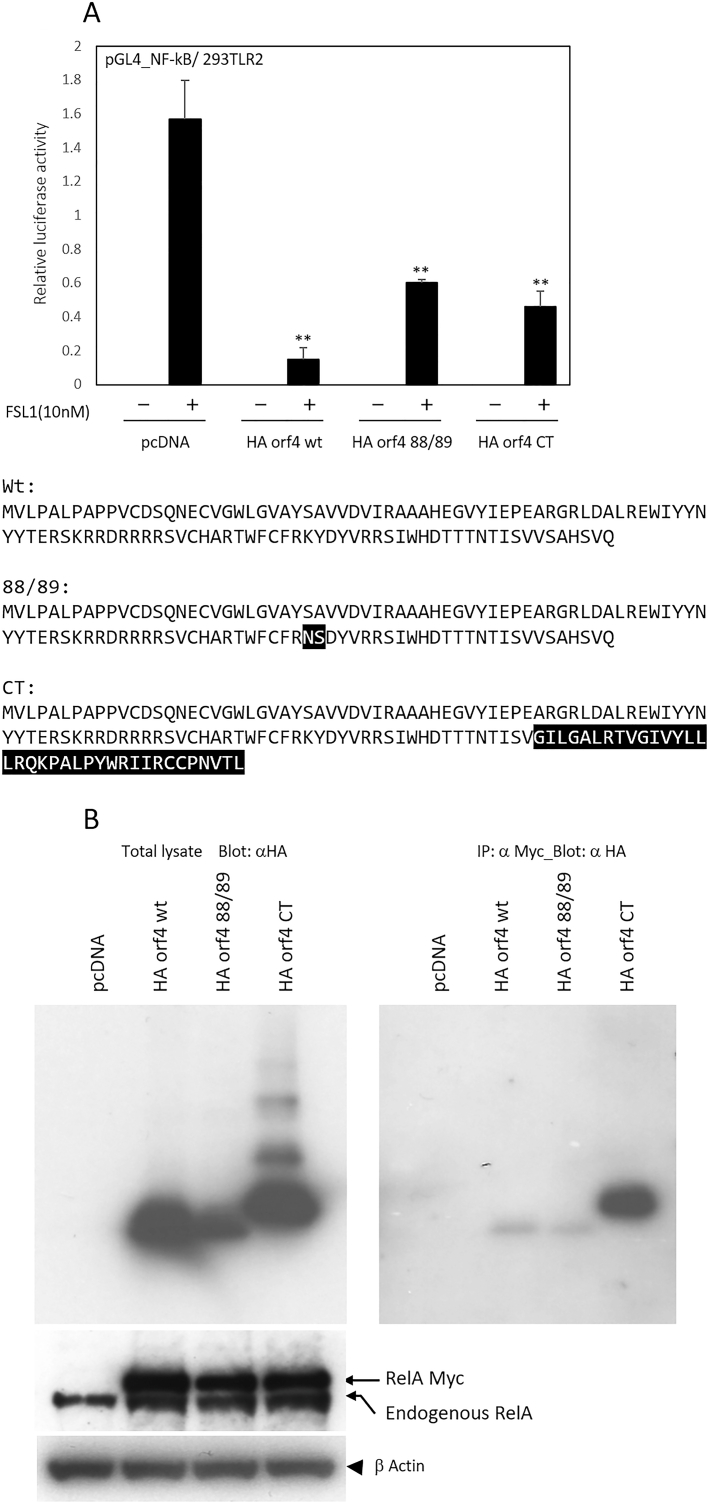
NF-κB repression by loss-of-PP2A-binding and a loss-of-killing mutant E4 orf4 mutant. A: 293TLR2 cells were transfected with either an E1 luciferase reporter plasmid or an NF-κB luciferase reporter plasmid, along with a pGL4.72 plasmid and the specified effector plasmids. The transfected cells were then incubated with FSL-1 (final concentration 10 nM) in serum-free medium for a duration of 6 h. Subsequently, a luciferase assay was conducted as previously described. The lower panel of the figure illustrates the amino acid sequences in E4 orf4 and its mutants. The C-terminal 36 amino acids of E4 orf4CT correspond to a frameshifted E4 orf6. The data presented are representative of three independent experiments. The error bars shown in the graphs indicate standard deviations calculated from triplicate wells within a single experiment. Comparisons were made using an unpaired Student's *t*-test. Results are represented as mean ± S.D. Statistical significance is displayed as: *, p < 0.05; * *, p < 0.01. These results are consistent across three separate independent experiments. B: HEK293 cells were transfected with the combination of myc-tagged RelA and indicated HA Eorf4s. Cells were incubated for 48 h and lysates were immunoprecipitated with anti-Myc antibody and blotted with anti-HA antibody. Exogenously expressed myc-tagged RelA has a larger molecular weight than endogenous RelA. The lowest row depicts anti-β Actin staining.

To investigate the association between candidate E4 orfs and RelA, typical NF-κB family protein, co-immunoprecipitation experiments were performed. As demonstrate in Fig. 2B, among E4 orf4, orf6, and orf 6/7, only E4 orf4 is pulldown with exogenously expressed RelA. Exogenously expressed myc-tagged RelA has a larger molecular weight than endogenous RelA.

### 3.3 Involvement of PP2A binding and killing responsible domain of E4 orf4 to NF-κB repression

Previous studies have demonstrated that E4 orf 4 induces TP53-independent cell death and virus spread via residues between the 51st and 89th amino acids [21]. To investigate the involvement of these phenotypes of E4 orf 4 in the repression of NF-κB, we cloned a loss-of-PP2A-binding and a loss-of-killing mutant (designated as E4 orf 88/89) as well as a C-terminus substitution mutant (E4 orf 4 CT). Luciferase reporter assays and co-immunoprecipitation experiments demonstrated that these mutations could not abolish the repressive effect and binding ability to human RelA (shown in Fig. 3A). Interestingly, the C-terminus substitution mutant (E4 orf 4 CT) exhibited a higher affinity with hRelA, but only a modest recovery of its repressive effect was observed when compared with wild-type E4 orf 4.

### 3.4 E4 orf 6/7 participated in NF-κB repression via E2Fs dependent pathways

**Fig. 4:**
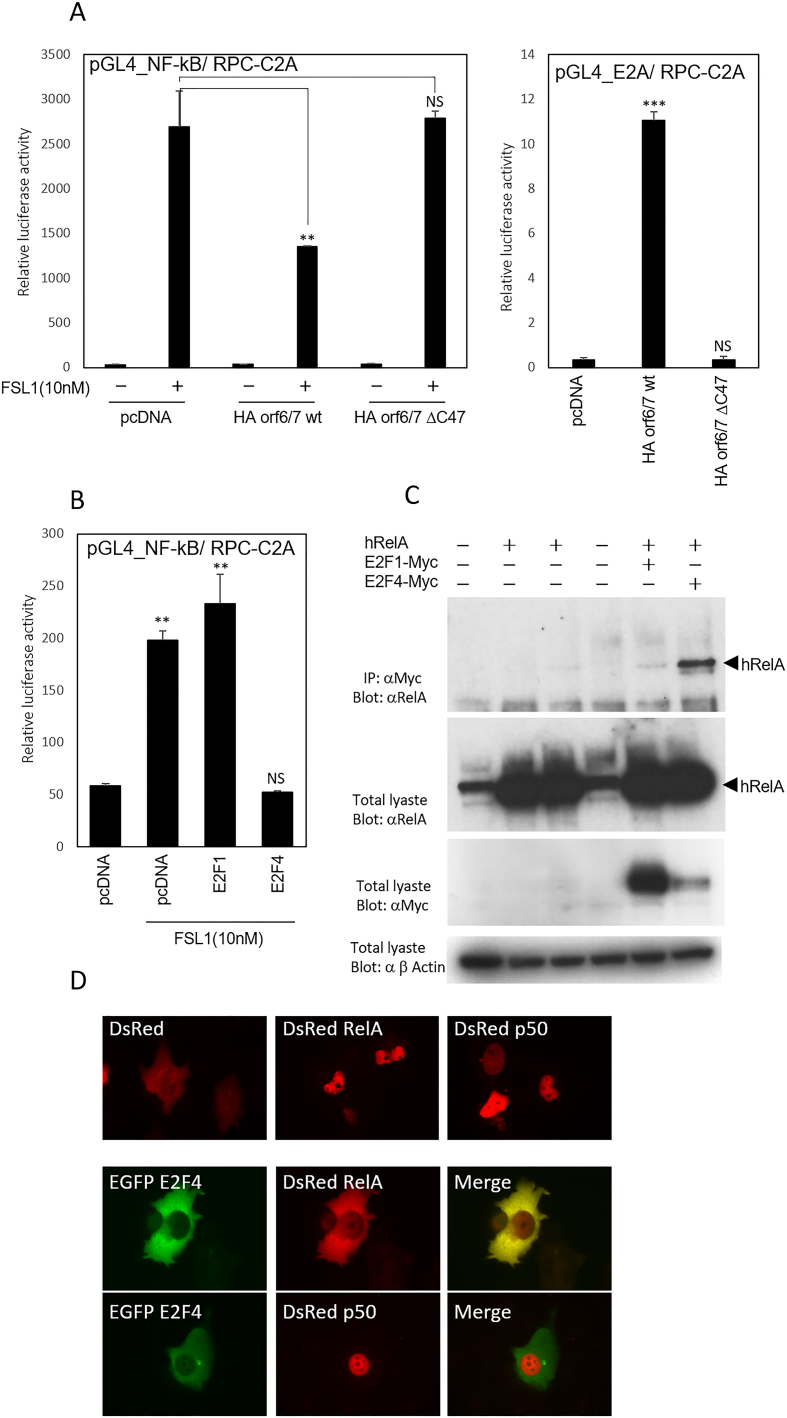
E4 orf6/7 repress NF-κB activation via E2F pathway. A: The indicated HA-tagged E4 orf6/7s and NF-κB luciferase reporter plasmid or E2A luciferase reporter, along with a pGL4.72 plasmid were transfected into RPC- C2A cells. Twenty-four hours after transfection, cells were incubated with FSL-1 (final concentration 10 nM) in serum-free medium for a duration of 6 h. Subsequently, a luciferase assay was conducted as previously described. The data presented are representative of three independent experiments. The error bars shown in the graphs indicate standard deviations calculated from triplicate wells within a single experiment. These results are consistent across three separate independent experiments. Comparisons were made using an unpaired Student's *t*-test. Results are represented as mean ± S.D. Statistical significance is displayed as: NS, not significant; *, p < 0.05; * *, p < 0.01. ; ***,p < 0.001. B: Myc-tagged E2F1 and E2F4, along with NF-kB luciferase reporter were transfected into RPC C2A cells. Twenty-four hours after transfection, cells were incubated with FSL-1 (final concentration 10 nM) in serum-free medium for a duration of 6 h. The data presented are representative of three independent experiments. The error bars shown in the graphs indicate standard deviations calculated from triplicate wells within a single experiment. Comparisons were made using an unpaired Student's *t*-test. Results are represented as mean ± S.D. Statistical significance is displayed as: NS, not significant; * *, p < 0.01. C: HEK293 cells were transfected with the combination of Myc tagged E2Fs and human RelA. Cells were incubated for 48 h and lysates were immunoprecipitated with anti-Myc antibody and blotted with anti-RelA antibody. The lowest row depicts anti-β Actin staining. D: GFP-tagged E2F4, together with DsRed-tagged RelA or DsRed-tagged p50, were co-transfected onto H1299 cells. After 24 h from transfection, cells were fixed with 4 % paraformaldehyde and observed by confocal laser scanning microscope.

Next, E4 orf6/7 and its C-terminus truncated mutant (ΔC47) were transfected into RPC-C2A along with NF-κB reporter, followed by FSL-1 stimulation. Fig. 4A demonstrates that the deletion of the C-terminus of E4 orf 6/7 is responsible for both NF-κB repression and E2F-dependent transcriptional activation. Given that it has been reported that E2F1 can associate with RelA [22], and E4 orf6/7 can stabilize the binding of E2Fs to their respective DNA sequences, we were encouraged to investigate the correlation between E2Fs and RelA. RPC C2A cells were transfected with NF-κB reporter and indicated combination of plasmids. Fig. 4B demonstrates that E2F4, but not E2F1, significantly inhibits the endogenously activated rat NF-κB-dependent transactivation of the luciferase reporter. Furthermore, the interaction between E2F4 and RelA was validated through a co-immunoprecipitation experiment (Fig. 4C).

It has been demonstrated that the most abundant form of canonical active NF-κB is heterodimer of RelA and NF-κB1/p50 [23]. We speculated that the complex formation between E2F4 and RelA might affect the subcellular colocalization of E2F4 and RelA. To confirm this possibility, GFP-tagged E2F4 was transfected together with DsRed-tagged RelA or DsRed-tagged NF-κB1. Under our experimental condition, cotransfected GFP-E2F4 and DsRed-RelA were colocalized in cytosol, whereas cotransfected GFP-E2F4 and DsRed-p50 did not indicate such a translocation (Fig. 4D).

## 4 Discussion

Toll-like receptors (TLRs) play a central role in innate immunity and are highly expressed in dendritic cells and macrophages [24]. Additionally, TLR2 is expressed ubiquitously in various tissues [25], therefore, TLR-dependent activation of NF-κB must occur at the entry site of the human adenovirus. Under our experimental condition, TLR2 were responsible for Ad recognition (Fig. 1B). Several studies have demonstrated that recombinant human adenovirus particles are potent inducers of inflammation and the resulting immune-mediated rejection of recombinant Ad [12,26]. The primary distinction between wild-type human adenoviruses and recombinant adenovirus vectors is the deletion of the E1 regions. The findings of our present study strongly suggest that E1-driven E4 proteins, most likely E4 orf4 and E4 orf6/7, suppress NF-κB activation at the beginning of the late infection phase (post-infection 24 h). The E4 promoter, located at least 180 bp upstream of the E4 transcription start site, contains several transcription factor target sequences, including E4F1 and ATFs. Transcription from the E4 start site is stimulated approximately 100-fold by E1A protein expression [27]. This may explain why the recombinant adenovirus, Ad-LacZ, could not repress NF-κB activation in RPC-C2A cells, but it could in HEK293 cells, which express endogenous E1A. Furthermore, it has been demonstrated that wild-type Ad (wt300) could repress NF-κB activation even in RPC C2A cells (Fig. 1B, C, and 1D).

E4orf4 is a 14 kDa protein consisting of 114 amino acids. It downregulates the expression of both cellular and viral genes induced by E1A. E4orf4 reduces AP-1 DNA binding activity, which is higher in cells infected with mutant Ads lacking E4orf4. This effect is attributed to decreased levels of JunB and c-Fos proteins, along with JunB mRNA. The Adenovirus E4orf4 protein reduces the phosphorylation of c-Fos and E1A proteins while simultaneously lowering the level of AP-1 [28]. E4 orf4 causes hypophosphorylation of E1A and c-Fos through its interaction with protein phosphatase 2A (PP2A). Inhibition of PP2A prevents the downregulation of JunB transcription by E4orf4. In this study, we found that E4orf4 binds with RelA/p65 and suppresses the endogenous activation of NF-κB via TLR2 (see Fig. 3B). On the other hand, our data suggest that the activation of NF-κB enhances E1 promoter activity (Fig. 2A). It has proposed that the C-terminal loop (residues 105–114) indicated great variability, and that residues known to be of importance to the interaction between E4orf4 and PP2A were not previously found within the E4orf4 C-terminus [29]. We observed that the C-terminal residues (108–114) did not participate in the association between E4 orf4 and RelA (Fig. 3B). However, the C-terminal substitution mutant of E4 orf4 (E4 orf4CT, C-terminal 36 amino acids correspond to a frameshifted E4 orf6) exhibited a significant suppressive effect and an enhanced binding affinity (Fig. 3A and B). This suggests that there are additional mechanisms contributing to the repression, rather than the direct binding with RelA. It is plausible that the accumulation of E4orf4 in the late phase of infection contributes to the diminished expression of E1A by inhibiting NF-κB activation.

E4orf4 has been shown to induce TP53-independent cell death [9,21]. Given that NF-κB family proteins play a pivotal role in promoting cellular survival in response to diverse challenges and stressors, it is conceivable that the interaction between E4orf4 and RelA might also play a role in the cell death function of E4orf4.

E4orf6/7 is expressed as a fusion of N-terminal E4orf6 and E4orf7 and dimerizes E2F factors, increasing the transactivation of the inverted E2F-responsive sites in the E2F1 and adenoviral E2 promoters [30]. We previously reported that E4orf6/7 induces apoptosis in cells in the presence of Tp53, and the C-terminal 47 amino acids of E4orf6/7 have been implicated in these functions [10]. In the present study, we have also revealed that the same C-terminal region is involved in the repression of TLR2-dependent NF-κB activation. Cook et al. reported that E1A repressed NF-κB -dependent transcription and sensitized cells to TNF-alpha induced apoptosis [31]. Tanaka et al. demonstrated the inhibitory function of E2F1 on NF-κB activation using the superoxide dismutase promoter. In their experimental conditions, they identified a physiological association between RelA/p65 and E2F1 through the dimerization domain of E2F1 using a yeast two-hybrid system [22]. In adenovirus-infected cells, physiological interaction between E1A proteins and Rb family proteins results in the dissociation of E2Fs from Rb family proteins and the activation of cellular genes containing an E2F-responsive element (TTTCGCGC), including the promoter region of Ad E2A [32]. Among the eight members of the E2F family, E2F1 through E2F3 are considered to be activators for E2F-dependent transcription, while E2F4 and E2F5 are considered to be transcriptional repressors [33]. The “activator” group of E2Fs is expressed maximally during the S phase of the cell cycle, whereas the “repressor” E2Fs are expressed abundantly throughout the cell cycle. Our current findings further demonstrate that E2F4 associates with RelA more effectively than E2F1 (shown in Fig. 4B and C) and the RelA/E2F4 complex was predominantly located at cytosol (Fig. 4D). It was also demonstrated that the C-terminal 118 amino acids of E2F4, which is not conserved in E2F1, are required for the interaction between E2F4 and RelA (data not shown). It seems likely that the sequestration of the RelA/E2F4 complex in the cytosol plays a main role in repressing NF-κB activation. It appears plausible that E4orf6/7 might contribute to the formation of the RelA-E2F4 complex via its C-terminal sequence, potentially cooperating with E1A.

In summary, this study demonstrated that two Ad E4 genes, E4orf4 and E4orf6/7, exhibit redundant repressive functions with NF-κB transactivation at 24 h post-infection. These repressive effects might contribute to the late phase of Ad replication. It is probable that these repressions serve as potent inducers of cell death; therefore, E4orf4 and orf6/7 should be retained in recombinant Ads used as oncolytic viruses rather than gene-transfer vectors.

## Funding

This work was supported by Grants-in-Aid for Science Research C-21K10130 provided by the 10.13039/501100001691Japan Society for the Promotion of Science.

## CRediT authorship contribution statement

**Anran Wang:** Investigation. **Kazuki Uchida:** Investigation. **Atsuro Yokoyama:** Methodology. **Fumihiro Higashino:** Methodology, Resources. **Motoaki Yasuda:** Supervision, Writing – review & editing.

## Declaration of competing interest

The authors declare that they have no known competing financial interests or personal relationships that could have appeared to influence the work reported in this paper.

## Figures and Tables

**Fig. S2:**